# Anti-CD74 antibodies have no diagnostic value in early axial spondyloarthritis: data from the spondyloarthritis caught early (SPACE) cohort

**DOI:** 10.1186/s13075-018-1535-x

**Published:** 2018-03-01

**Authors:** Janneke J. de Winter, Marleen G. van de Sande, Niklas Baerlecken, Inger Berg, Roberta Ramonda, Désirée van der Heijde, Floris A. van Gaalen, Torsten Witte, Dominique L. Baeten

**Affiliations:** 1 Department of Clinical Immunology and Rheumatology, Amsterdam Rheumatology and immunology Center, Academic Medical Center/University of Amsterdam, Amsterdam, The Netherlands; 2 Department of Immunology and Rheumatology, Medical University Hannover, Hannover, Germany; 3 Department of Rheumatology, Diakonhjemmet Hospital, Oslo, Norway; 4 Rheumatology unit, Department of medicine, University of Padua, Padua, Italy; 5 Department of Rheumatology, Leiden University Medical Center, Leiden, The Netherlands; 6 Private Practice, Cologne, Germany

**Keywords:** Axial spondyloarthritis, Early diagnosis, Biomarker

## Abstract

**Background:**

Anti-CD74 IgG antibodies are reported to be elevated in patients with axial spondyloarthritis (axSpA). This study assessed the diagnostic value of anti-CD74 antibodies in patients with early axSpA.

**Methods:**

Anti-CD74 IgG and IgA antibodies were first measured in an exploratory cohort of patients with radiographic axSpA (138 patients with ankylosing spondyloarthritis (AS)) and 57 healthy controls and then were measured in patients with early axSpA (*n* = 274) and with non-SpA chronic back pain (CBP) (*n* = 319), participating in the spondyloarthritis caught early (SPACE) prospective cohort study of patients under 45 years old with early back pain (for ≥ 3 months, but ≤ 2 years).

**Results:**

In the exploratory cohort, anti-CD74 IgG antibodies were present in 79.7% of patients with AS vs. 43.9% of healthy controls (*p* < 0.001). Anti-CD74 IgA antibodies were present in 28.5% of patients with AS vs. 5.3% of healthy controls (*p* < 0.001). In the SPACE cohort, anti-CD74 IgG antibody levels were present in 46.4% of the patients with axSpA vs. 47.9% of the patients with CBP (*p* = 0.71). Anti-CD74 IgA antibodies were present in 54.7% of the patients with axSpA and 37.0% of the patients with CBP (*p* < 0.001). This resulted in a positive predictive value of 58.8% (compared to a prior probability of 46.2%) and a negative predictive value of 59.1% (compared to a prior probability of 53.8%). In a regression model, total serum IgA was associated with axSpA odds ratio (OR) 1.19, *p* < 0.001) whereas anti-CD74 IgA was not (OR) 1.01, *p* = 0.33). Furthermore, anti-CD74 IgA was associated with sacroiliitis on magnetic resonance imaging (MRI) (OR) = 2.50, *p* = 0.005) and heel enthesitis (OR) = 2.56, *p* = 0.002).

**Conclusions:**

Albeit anti-CD74 IgA is elevated in patients with early axSpA, this elevation is not sufficiently specific to yield significant diagnostic value in patients under 45 years old presenting with early back pain.

## Background

Axial spondyloarthritis (axSpA) is a prevalent form of chronic inflammatory arthritis, affecting 0.5–1.5% of the Western population [1, 2]. Current biological disease markers in axSpA (human leukocyte antigen (HLA)-B27, C-reactive protein (CRP), sacroiliitis shown on radiography or magnetic resonance imaging (MRI)) have insufficient diagnostic properties to rely on for making a diagnosis, impeding early diagnosis and treatment. Although diagnostic delays have decreased over recent years as a result of modern imaging techniques and more awareness of axSpA, the current diagnostic delay ranging from 5 to 10 years [3–6] prevents treatment in the early disease phase.

Two recent studies provided preliminary evidence that anti-CD74 antibodies are elevated in SpA [7, 8]. CD74, also known as the HLA class II γ-chain or invariant chain, is involved in the assembly of major histocompatibility complex II and in preventing premature peptide binding [9]. The extracellular part has two different domains, thyroglobulin type-1 and class II-associated invariant chain peptide (CLIP). Binding of antibodies to CD74 may lead to activation of cells and production of proinflammatory cytokines such as tumor necrosis factor α (TNFα) [10]. The first cross-sectional study showed that anti-CD74 IgG antibodies were present in 67% of the 216 tested patients with axSpA, compared to 6% of 285 non-SpA controls (blood donors or patients with diseases other than axSpA) [8]. The second study showed that IgG anti-CD74 antibodies were detected in 85% of the 145 patients with axSpA and in 8% of the 51 patients without SpA [7].

Although indicating the potential importance of anti-CD74 IgG antibodies in axSpA, the role of anti-CD74 antibodies in a diagnostic setting is still not confirmed. Therefore, the aim of this study was to test the level and diagnostic value of anti-CD74 IgG and IgA antibodies in a “real life” diagnostic setting using patients with axSpA and patients with chronic back pain (CBP) from a cohort of patients with early back pain, the spondyloarthritis caught early (SPACE) cohort.

## Methods

### Exploratory cohort: patients with radiographic axSpA and healthy controls

Serum from patients with radiographic axSpA (ankylosing spondyloarthritis (AS)) was collected in the Academic Medical Center/University of Amsterdam (*n* = 21) and the Medical University of Hannover (*n* = 117). All patients with AS were diagnosed by a rheumatologist with AS and fulfilled the modified New York (mNY) criteria for AS. Serum from healthy controls was collected in the Academic Medical Center/University of Amsterdam (*n* = 19) and the Medical University of Hannover (*n* = 38). All patients with AS and all healthy controls gave their written informed consent and the studies were approved by the local ethics committees of the Academic Medical Center/University of Amsterdam and the Medical University of Hannover. Serum samples were stored for at least 6 months at − 80 °C.

We included 138 patients with AS and 57 healthy controls. The mean (SD) age was 46.1 (13.1) in patients with AS and 36.7 (13.5) in healthy controls (*p* < 0.001). Of the patients with AS, 92 out of 117 were male vs. 22 out of the 38 healthy controls (78.6% vs. 57.9%, *p* = 0.012). HLA-B27 was determined in 117 out of 138 patients with AS; 108 out of 117 patients were HLA-B27-positive (92.3%). The mean disease duration was 24.3 (13.2) months.

### The SPACE cohort

We used baseline serum samples from the SPACE cohort. The SPACE cohort is an ongoing, prospective, multicenter, longitudinal cohort that started in 2009 and has previously been described in detail [11]. In short, the SPACE cohort includes patients aged ≥ 16 years with chronic back pain (for ≥ 3 months, but ≤ 2 years) with onset before the age of 45 years. The local medical ethical committees of the participating sites approved the study and all participants gave their written informed consent. For the present analyses, data and serum from the baseline visit were used. We used the central reading results of radiography and MRI of the sacroiliac joints [11]. The primary outcome was an axSpA diagnosis by a rheumatologist vs. no axSpA diagnosis by a rheumatologist. As secondary outcomes, we used (1) a strict definition of axSpA: patients diagnosed by a rheumatologist as having axSpA plus fulfilling the Assessment of SpondyloArthritis International Society (ASAS) axSpA criteria vs. patients not diagnosed by a rheumatologist as having axSpA and not fulfilling the ASAS axSpA criteria, and (2) patients participating in SPACE diagnosed by a rheumatologist as having axSpA at baseline and after 1 year of follow up vs. patients not diagnosed as axSpA on assessment at these two time points. Serum samples were collected at baseline and stored for at least 6 months at − 80 °C.

We included 593 patients from the SPACE cohort, including 274 patients diagnosed with axSpA at baseline. Table 1 shows baseline characteristics of the study patients. In short, 119 of the patients with axSpA were male vs. 81 of the patients with CBP (43.4% vs. 28.3%, *p* < 0.001). The mean age of the patients with axSpA was 30.7 (7.9) years at inclusion and in the patients with CBP it was 31.4 (8.5) years (*p* = 0.83). Of the axSpA patients, 165 were HLA-B27-positive versus 63 of the patients with CBP (60.7% vs. 22.1%, *p* < 0.001). Of the patients with axSpA, 172 fulfilled the ASAS axSpA criteria and 48 of the patients with CBP fulfilled the ASAS axSpA criteria (62.8% vs. 37.2%, *p* < 0.001).

**Table 1:** Demographic and disease characteristics of patients with axial spondyloarthritis (axSpA) and patients with chronic back pain (CBP) participating in the SPACE cohort

	Diagnosed with axSpA		
	Yes (*n* = 274)	No (*n* = 286)	*P* value
Male (*n*, %)	119 (43.4)	181 (28.3)	<0.001
Age (mean, SD)	30.7 (7.9)	31.4 (8.5)	0.83
HLA-B27 positive (*n*, %)	165 (60.7)	63 (22.1)	<0.001
Positive family history (*n*, %)	131 (47.8)	111 (38.8)	0.03
Duration of back pain in months (mean, SD)	13.1 (7.0)	13.5 (7.2)	0.59
Inflammatory back pain (*n*, %)	213 (77.7)	154 (54.0)	<0.001
Peripheral arthritis (*n*, %)	59 (21.6)	23 (8.1)	<0.001
Enthesitis (*n*, %)	91 (33.2)	21 (7.3)	<0.001
Dactylitis (*n*, %)	25 (9.1)	4 (1.4)	<0.001
IBD (*n*, %)	21 (7.7)	16 (5.6)	0.32
Psoriasis (*n*, %)	43 (15.7)	19 (6.6)	0.001
Uveitis (*n*, %)	32 (11.7)	7 (2.4)	<0.001
Tender joint count (mean, SD)	2.2 (5.0)	1.9 (3.7)	0.38
Swollen joint count (mean, SD)	0.6 (2.1)	0.1 (0.6)	0.001
CRP, mg/L (median, IQR)	3.0 (3.0–5.0)	3.7 (3.0–7.0)	0.001
CRP >5 mg/L (*n*, %)	113 (41.7)	70 (26.2)	<0.001
ESR, mm/h (mean, SD)	14.9 (15.8)	9.2 (11.4)	<0.001
ESR >20 mm/h (*n*, %)	63 (23.6)	20 (7.1)	<0.001
ASAS axSpA criteria (*n*, %)	172 (62.8)	48 (16.8)	<0.001
mNY criteria (*n*, %)	33 (14.4)	5 (2.0)	<0.001
Any DMARD use (*n*, %)	38 (14.0)	14 (5.1)	<0.001
NSAID use (*n*, %)	197 (72.4)	172 (60.8)	0.004
BASDAI (mean, SD)	4.2 (2.1)	4.7 (2.0)	0.01
Diagnosed with axSpA at 1-year follow up (*n*, %)	143 (52.2)	16 (5.6)	<0.001

Missing values were below 5% except for axial spondyloarthritis (axSpA) diagnosis (5.6%), SJC (5.7%), C-reactive protein (CRP) (7.1%), modified New York (mNY) criteria (17.3%), Bath Ankylosing Spondylitis Disease Activity Index (BASDAI) (15.0%) and the number of patients still diagnosed as having axSpA after 1 year of follow up (58.9%)

*ASAS* Assessment of SpondyloArthritis International Society, *DMARD* disease-modifying antirheumatic drug, *ESR* erythrocyte sedimentation rate, *IBD* inflammatory bowel disease, *NSAID* nonsteroidal anti-inflammatory drug

Missing values were below 5% except for axSpA diagnosis (5.6%), swollen joint count (SJC (5.7%)), C-reactive protein (CRP (7.1%)), sacroiliitis according to the modified New York (mNY) criteria (17.3%), Bath Ankylosing Spondylitis Disease Activity Index (BASDAI) (15.0%)), total serum IgA (9.2%) and the number of patients missing the 1-year evaluation (58.9%). This was mostly due to the study protocol: not all patients were invited for follow up and some patients did not have a 1-year visit due to shorter follow up.

### Anti-CD74 antibody detection

Two different enzyme-linked immunosorbent assays (ELISAs) were performed to detect anti-CD74 IgG and anti-CD74 IgA antibodies. The Medical University of Hannover developed the ELISAs to detect anti-CD74 IgG and IgA antibodies in cooperation with AESKU Diagnostics (Wendelsheim, Germany), as described earlier by Baerlecken et al. [8]. The originally described ELISAs were improved for the current study as the old (peptide-based) test only worked with serum that had been frozen for a long time. The tests were performed according to the manufacturer’s protocol. Anti-CD74 IgG is expressed as optical density (OD), anti-CD74 IgA is expressed as OD (AS vs. healthy controls) or U/mL (in the SPACE cohort, at that time standard serum was available). We also measured the amount of total serum IgA, as earlier studies showed that total serum IgA is elevated in axSpA [12, 13].

### Data analysis

The chi-square (χ2) test and Mann-Whitney U test were used for categorical and continuous data, respectively. Categorical data are presented as number (percent), continuous data are presented as the mean (SD) or as median (interquartile range (IQR)) as appropriate. Statistical tests were two-sided, and *p* values < 0.05 were considered significant. Only the available data were analyzed. Linear correlation between two continuous variables was calculated by calculating Pearson’s correlation coefficient (*r*). Receiver operating characteristic (ROC) analysis and the maximum value of the Youden index (sensitivity + specificity – 1) [14] was used to evaluate the predictive value of anti-CD74 IgG and IgA antibodies and to calculate the best possible cutoff. These cutoff values were used to calculate the positive predictive value (PPV) and negative predictive value (NPV) and positive and negative likelihood ratios (LR+ and LR-, respectively) of anti-CD74 IgG and IgA antibodies in discriminating between patients with axSpA and patients with CBP.

We explored which characteristics among patients with axSpA were associated with higher anti-CD74 IgA levels by logistic regression. We started with a set of predetermined candidate variables: HLA-B27, disease duration, sacroiliitis on radiography or MRI, peripheral (arthritis, dactylitis and heel enthesitis) and extra-articular (uveitis, psoriasis and inflammatory bowel disease (IBD)) disease manifestations and used backward elimination to create the final model.

We evaluated the association between anti-CD74 IgA and total IgA by univariate and multivariate logistic regression by both forward selection and backward elimination. Besides total IgA and anti-CD74 IgA we added potential confounding variables: HLA-B27, disease duration, sacroiliitis on radiography or MRI, peripheral (arthritis, dactylitis and heel enthesitis) and extra-articular (uveitis, psoriasis and IBD) disease manifestations. Since the interpretation of the regression analysis might be influenced by collinearity between anti-CD74 and total IgA, we first assessed the amount of collinearity by calculating Pearson’s correlation coefficient, variance inflating factor (VIF) and tolerance (*R*^2^). Values of Pearson’s correlation coefficient >0.7, VIF >5 and tolerance <0.20 were considered problematic for interpreting the regression model [15]. We used IBM SPSS Statistics 24 for all analyses.

## Results

### Anti-CD74 IgG antibodies

To confirm the earlier results [7, 8], we first measured the level, prevalence and diagnostic value of anti-CD74 IgG antibodies. In the exploratory cohort, median anti-CD74 IgG antibodies (OD) were higher in patients with AS than in healthy controls (0.70 vs. 0.51, *p* < 0.001). The AUC on ROC analysis was 0.70 (95% CI 0.62–0.79) and resulted in a cutoff of 0.52. Using this cutoff, anti-CD74 IgG antibodies were present in 110 out of 138 patients with AS vs. 25 out of 57 healthy controls (79.7% vs. 43.9%, *p* < 0.001).

In the SPACE cohort, median anti-CD74 IgG antibodies (OD) did not differ between patients with axSpA and patients with CBP (0.50 vs. 0.52, *p* = 0.15). The AUC on ROC analysis was 0.47 (95% CI 0.42–0.51). Using a cutoff of 0.52, anti-CD74 IgG antibodies were present in 127 out of 274 patients with axSpA and in 137 out of 286 patients with CBP (46.4% vs. 47.9%, *p* = 0.71) resulting in a PPV of 48.1%, a NPV of 50.3%, a LR+ of 0.97 and a LR- of 1.03.

### Anti-CD74 IgA antibodies

Because anti-CD74 IgG antibodies did not have diagnostic value in the SPACE cohort, we explored the level, prevalence and diagnostic value of anti-CD74 IgA antibodies, as earlier research shows that total serum IgA is elevated in axSpA [12, 16] and that IgA is produced at mucosal surfaces, including the gut, which may be inflamed in spondyloarthritis [17].

**Fig. 1:**
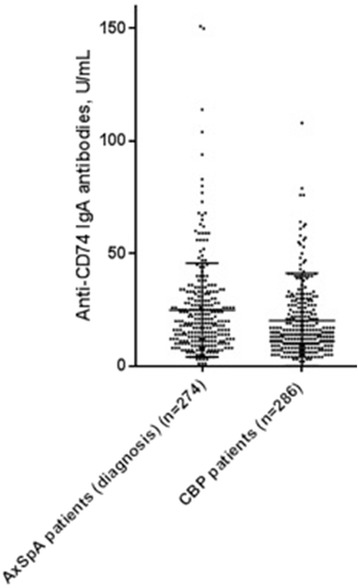
Anti-CD74 IgA antibody levels measured in serum from participants in the SPACE cohort. Axial spondyloarthritis (axSpA) (diagnosis), participants in SPACE who were diagnosed with axSpA by a rheumatologist at baseline; CBP, patients with chronic back pain. One data point is outside the axis limits

In the exploratory cohort, median anti-CD74 IgA antibodies (OD) were higher in patients with AS than in healthy controls (0.31 vs. 0.20, *p* < 0.001). The AUC on ROC analysis was 0.78 (95% CI 0.82–0.79) and resulted in a cutoff of 0.22 (Fig. 1). Using this cutoff, anti-CD74 IgA antibodies were present in 39 of 138 patients with AS and in 3 out of 57 healthy controls (28.5% vs. 5.3%, *p* < 0.001.

In the SPACE cohort, median anti-CD74 IgA antibodies (U/mL) were higher in patients with axSpA than in patients with CBP (19.9 vs. 14.0, *p* < 0.001, Fig. 1). The AUC on ROC analysis was 0.60 (95% CI 0.55–0.65). Using a cutoff of 18.0 U/mL, anti-CD74 IgA antibodies were positive in 150 of 274 patients with axSpA vs. 105 of 284 patients with CBP (54.7% vs. 37.0%, *p* < 0.001). Anti-CD74 IgA testing with a pre-test probability of axSpA of 46.2% (and thus 53.8% not having axSpA) resulted in a PPV of 58.8%, a NPV of 59.1%, a LR+ of 1.48 and a LR- of 0.72.

### Analyses using different definitions of axSpA

As the results of the diagnostic biomarker analysis may depend on the “gold standard” for diagnosis of axSpA, we also tested the median anti-CD74 IgG and IgA levels looking at (1) patients in the SPACE cohort who were diagnosed using a strict definition of axSpA (diagnosed as by a rheumatologist and also according to the Assessment of SpondyloArthritis International Society (ASAS) criteria) (*n* = 172) versus those not fulfilling either rheumatologist diagnosis or ASAS criteria (*n* = 238) and (2) patients in the SPACE cohort with confirmed diagnosis of axSpA (*n* = 143) or no axSpA (*n* = 64) after 1 of year follow up (1 year follow-up data were available in 244 patients in total). As shown in Table 2, the results of these additional analyses were comparable to the primary analyses.

**Table 2:** Anti-CD74 antibody and IgA levels in serum from patients in the SPACE cohort

	AxSpA (diagnosis)			Diagnosis and ASAS axSpA criteria			AxSpA (diagnosis) confirmed at 1-year follow up		
	Yes (*n* = 274)	No (*n* = 286)	*P* value	Yes (*n* = 172)	No (*n* = 238)	*P* value	Yes (*n* = 143)	No (*n* = 64)	*P* value
Anti-CD74 IgG (OD)	0.50 (0.32–0.65)	0.52 (0.38–0.69)	0.15	0.50 (0.30–0.67)	0.52 (0.39–0.70)	0.075	0.43 (0.30–0.58)	0.48 (0.35–0.61)	0.32
Anti-CD74 IgA (U/mL)	19.9 (11.4–30.6)	14.0 (9.3–26.0)	<0.001	21.8 (11.6–31.9)	13.5 (8.4–22.3)	<0.001	17.6 (9.9–29.0)	13.7 (9.4–23.6)	0.10
Total IgA (g/L)	4.07 (2.90–4.97)	3.37 (2.57–4.66)	<0.001	4.06 (3.01–5.03)	3.29 (2.42–4.62)	0.001	4.02 (2.77–4.78)	3.66 (2.43–4.74)	0.14

Results are shown as median (IQR). Axial spondyloarthritis (axSpA) (diagnosis): SPACE participants diagnosed with axSpA by a rheumatologist at baseline; Assessment of SpondyloArthritis International Society (ASAS) axSpA criteria: SPACE participants fulfilling versus not fulfilling the ASAS axSpA criteria at baseline; Diagnosis and ASAS axSpA: SPACE participants diagnosed as having axSpA and fulfilling the ASAS axSpA criteria versus patients not diagnosed as having axSpA and not fulfilling the ASAS axSpA criteria. Missing values were 5.6% for diagnosis of axSpA, 3% for fulfilling the ASAS axSpA criteria, 30.9% for diagnosis and ASAS axSpA criteria and 58.9% for axSpA diagnosis at 1-year follow up

*OD* optical density

### Anti-CD74 IgA antibodies in axSpA subpopulations

We additionally explored if anti-CD74 IgA antibodies were associated with a specific sub-population of patients with axSpA. In the exploratory cohort, the mean disease duration in patients with AS with or without anti-CD74 IgA antibodies did not differ (24.12 vs. 24.35 months, respectively, *p* = 0.931).

In the SPACE cohort, on comparison of patients with axSpA with or without HLA-B27, elevated CRP, inflammatory back pain, sacroiliitis shown on MRI and peripheral (enthesitis, arthritis or dactylitis) or extra-articular (psoriasis, uveitis or IBD) manifestations there were no differences (data not shown). Patients with axSpA with radiographic sacroiliitis more often had anti-CD74 IgA antibodies than patients with axSpA without radiographic sacroiliitis (66.7% vs. 46.4%, *p* = 0.031). In regression analysis within the population of patients with axSpA, sacroiliitis on MRI and heel enthesitis were both significantly associated with elevated anti-CD74 IgA odds ration (OR) = 2.50, *p* = 0.005 and (OR) = 2.56, *p* = 0.002, respectively). HLA-B27, CRP, inflammatory back pain, radiographic sacroiliitis and peripheral manifestations other than heel enthesitis or extra-articular manifestations were not associated with anti-CD74 IgA levels.

### Total serum IgA

Since earlier studies showed that total serum IgA is elevated in patients with AS [12, 13], we additionally measured total serum IgA in the SPACE cohort. The AUC on ROC curve analysis was 0.59. Total IgA was higher in patients with axSpA than in patients with CBP (4.07 vs. 3.37 g/L, *p* < 0.001, Table 2). Total serum IgA was higher than the upper reference range (4.0 g/L) in 49.0% of the patients with axSpA and in 35.8% of the patients with CBP (*p* = 0.008). Anti-CD74 IgA and total IgA were both statistically significant predictors of diagnosis of axSpA, in a univariate logistic regression model (data not shown). We tested for collinearity by first measuring Pearson’s correlation coefficient, which was 0.31 for total IgA and anti-CD74 IgA (*p* < 0.001). Second, we determined the VIF in a linear regression model, showing that the amount of collinearity was low (VIF of 1.11 and *R*^2^ of 0.90). In a multivariate logistic regression model including anti-CD74 IgA and total IgA, total serum IgA was associated with a diagnosis of axSpA (OR) 1.19, *p* < 0.001), whereas anti-CD74 IgA was not (OR) 1.01, *p* = 0.33). Adding other potential confounding variables to the model (CRP, inflammatory back pain, HLA-B27, inflammatory bowel disease) did not change the results.

## Discussion

The results of our study suggest that (1) serum anti-CD74 IgG and IgA antibodies are elevated in patients with AS compared to healthy controls, (2) serum anti-CD74 IgG antibodies are not elevated in patients with early axSpA compared to patients with CBP, (3) serum anti-CD74 IgA antibody levels are elevated in patients with early axSpA compared to patients with CBP, but (4) serum anti-CD74 IgA antibodies have no diagnostic value in patients with early back pain because of small numerical differences.

Previous studies showed that anti-CD74 IgG antibodies are present in 69% and 85% of patients with (mostly radiographic) axSpA [7, 8] in comparison with 6% and 8% of individuals in control populations without axSpA or back pain. Somewhat lower numbers were obtained in the current study when assessing patients with radiographic axSpA in the SPACE cohort, as 54.5% had anti-CD74 IgG antibodies (data not shown). However, our study revealed two novel pieces of information. First, the percentage of patients with non-radiographic axSpA who were positive for anti-CD74 IgG antibodies was lower (43.9%), suggesting that either the antibodies develop over time or that they are associated with radiographic sacroiliitis rather than with the diagnosis of axSpA per se. Second, the percentage of control patients with elevated anti-CD47 IgG antibodies (47.9%) was much higher in our study than in the previous reports. This may be due either to the fact that the antibodies are higher in patients with CBP than in healthy individuals, or may be explained by the fact that the anti-CD74 IgG assay has been modified over time. Indeed, the original assay was sensitive to interference by soluble CD74 in serum, leading to false negative results in samples where sCD74 had not been degraded by freezing-thawing. In contrast, the assay used here is not biased by the presence of sCD74 and should therefore be more reliable.

In contrast with the previous suggestion that the presence of anti-CD74 IgG was especially high in early axSpA and declined with longer disease duration [7, 8], disease duration did not correlate with anti-CD74 IgG but we noted lower presence in non-radiographic axSpA and higher presence in controls with CBP. Collectively, these data indicate that anti-CD74 IgG has no significant diagnostic value in patients under 45 years old presenting with recent-onset back pain.

A second important finding of this study is that anti-CD74 IgA appear better that anti-CD74 IgG antibodies as a biomarker in axSpA. In contrast to IgG, the anti-CD74 IgA antibody levels were indeed higher in patients with axSpA than in patients with CBP. Another study investigating the sensitivity and specificity of anti-CD74 antibodies in a cohort of patients with axSpA (the InterSpA cohort) compared to healthy donors showed that anti-CD74 IgA antibodies were elevated in 65.4% of 104 patients fulfilling the ASAS axSpA criteria [18]. When we used the same cutoff as was used in the InterSpA cohort (15 U/mL), 63.1% of the SPACE patients with axSpA had elevated anti-CD74 IgA antibodies. However, 47.5% of the patients with CBP also had elevated anti-CD74 IgA antibodies when using this cutoff. Despite the numerical differences in the SPACE cohort between patients with axSpA and CBP, testing for anti-CD74 IgA antibodies only modestly increased the pre-test probability of axSpA (46.2%) to 58.8% post-test probability. Similarly, the NPV of anti-CD74 IgA antibodies was low. The LR+ of 1.48 and LR- of 0.72 confirmed that although anti-CD74 is associated with axSpA, their practical significance in diagnostic testing is low. Moreover, the association between axSpA and anti-CD74 IgA antibodies in early back pain may partly be explained by total IgA levels, which have previously been shown to be elevated in patients with axSpA [12, 13]. In the SPACE cohort, total IgA was significantly correlated with CRP and ESR levels (data not shown), showing that IgA levels might be linked to the level of inflammation, which is in line with previous research [19, 20]. In multivariate analysis, the value of anti-CD74 IgA disappeared when adding total IgA levels.

The strengths of our study include the fact that the SPACE cohort is a well-validated “real life”, multicenter, multinational cohort with large numbers of patients. The entry criteria for SPACE were designed to reflect at best the clinical practice of patients presenting with chronic back pain to a rheumatologist and in which the rheumatologist considers the possibility of axSpA in his/her differential diagnosis. The work-up of these patients in the SPACE cohort is very similar to what one would do in clinical practice. As such, almost all patients in the SPACE cohort considered as having axSpA (and as indicated (Table 2), to be even more stringent we used not only clinical diagnosis as a reference but also the combination of clinical diagnosis plus diagnosis according to the ASAS axSpA criteria) would also be considered as having SpA in daily practice. Supporting our claim, the percentage of patients with axSpA in SPACE (42% when using both clinical diagnosis and ASAS axSpA criteria) is similar to the percentage of 41.8% in a multicenter study using a referral strategy consisting of the presence of either IBP or HLA-B27 or sacroiliitis on imaging (MRI and/or radiography) [21] and not much higher than the 35.1% in a study using IBP (inflammatory back pain) or a good non-steroidal anti-inflammatory drug (NSAID) response as referral symptom [22]. So percentages around 40% are in line with what one would expect in such “diagnostic” settings.

Furthermore, the meticulous collection of clinical and radiographic data enabled us to define axSpA in several ways and thus correct for potential biases. For example, an accurate clinical diagnosis of axSpA in an early phase remains challenging and the ASAS classification criteria are not appropriate for diagnostic use, questioning the validity of the gold standard for assessment of a potential diagnostic biomarker. The SPACE cohort, however, allowed us to use more strict definitions of diagnosis, either by combining clinical diagnosis and classification criteria or by using clinical diagnosis confirmed at 1 year of follow up. These analyses yielded very similar results to the primary analyses, indicating that it is unlikely that diagnostic biases explain the differences in anti-CD74 IgG between established AS and early axSpA, the relatively high prevalence of anti-CD74 IgG and IgA antibodies in patients with CBP, or the limited PPV and NPV of anti-CD74 IgA in patients with early back pain.

Similarly, the well-documented clinical and imaging data from the SPACE cohort also allowed us to explore if anti-CD74 IgA antibodies were associated with a specific sub-population of patients with axSpA. These analyses showed that anti-CD74 IgA antibodies were not associated with HLA-B27, CRP, disease duration, sacroiliitis on radiography, peripheral manifestations other than heel enthesitis or extra-articular disease manifestations, although they were associated with sacroiliitis on MRI and with heel enthesitis. A longitudinal analysis may be more powerful than our cross-sectional approach to determine whether anti-CD74 antibodies are a useful biomarker for specific features of the disease, for example, to predict radiographic damage.

Although the SPACE cohort is designed to simulate a real-life situation, the generalizability of findings in the SPACE cohort to the intended population could be questioned. First, the SPACE cohort includes patients with chronic back pain for ≥ 3 months but ≤ 2 years, between 16 and 45 years old, in whom the diagnosis of AxSpA was considered. However, patients presenting for diagnostic work-up of potential AxSpA in clinical practice may have significantly longer symptom duration. Although we did not find any correlation between anti-CD74 IgG or IgA antibodies and disease duration in either of our cohorts, we cannot formally exclude a different relationship in patients with longer symptom duration. Second, the SPACE cohort includes patients in secondary and tertiary rheumatology practices, so this is the setting that we tested, and our data are not applicable to a primary care or population-based setting. In the latter settings, however, the pretest probability would be much lower and consequently, the performance of the test would even be worse.

## Conclusion

We conclude that anti-CD74 IgG and IgA antibodies are of limited value in diagnosing axSpA in patients with early, chronic back pain. Long-term follow up of patients in the SPACE cohort will show whether anti-CD74 IgA antibodies contribute to predicting certain axSpA characteristics such as radiographic damage, extra-articular manifestations or peripheral joint disease.

## References

[1] Dougados M, Baeten D (2011). Spondyloarthritis. Lancet..

[2] Reveille JD, Witter JP, Weisman MH (2012). Prevalence of axial spondylarthritis in the United States: estimates from a cross-sectional survey. Arthritis Care Res (Hoboken)..

[3] Sykes MP, Doll H, Sengupta R, Gaffney K (2015). Delay to diagnosis in axial spondyloarthritis: are we improving in the UK?. Rheumatol (Oxford)..

[4] Feldtkeller E, Rudwaleit M, Zeidler H (2013). Easy probability estimation of the diagnosis of early axial spondyloarthritis by summing up scores. Rheumatology (Oxford)..

[5] Masson Behar V, et al. Diagnostic delay in axial spondyloarthritis: a cross-sectional study of 432 patients. Joint Bone Spine. 2016; 10.1016/j.jbspin.2016.06.005. 10.1016/j.jbspin.2016.06.005 27450199

[6] Rudwaleit M, van der Heijde D, Khan MA, Braun J, Sieper JC (2004). How to diagnose axial spondyloarthritis early. Ann Rheum Dis..

[7] Baraliakos X, Baerlecken N, Witte T, Heldmann F, Braun J (2014). High prevalence of anti-CD74 antibodies specific for the HLA class II-associated invariant chain peptide (CLIP) in patients with axial spondyloarthritis. Ann Rheum Dis..

[8] Baerlecken NT (2014). Autoantibodies against CD74 in spondyloarthritis. Ann Rheum Dis..

[9] Neefjes J, Jongsma ML, Paul P, Bakke O (2011). Towards a systems understanding of MHC class I and MHC class II antigen presentation. Nat Rev Immunol..

[10] Starlets D (2006). Cell-surface CD74 initiates a signaling cascade leading to cell proliferation and survival. Blood..

[11] van den Berg R (2013). Percentage of patients with spondyloarthritis in patients referred because of chronic back pain and performance of classification criteria: experience from the Spondyloarthritis Caught Early (SPACE) cohort. Rheumatology (Oxford)..

[12] Cowling P, Ebringer R, Ebringer A (1980). Association of inflammation with raised serum IgA in ankylosing spondylitis. Ann Rheum Dis..

[13] Wendling D (1994). Spondylarthropathies and the IgA system. Rev Med Interne..

[14] Youden WJ (1950). Index for rating diagnostic tests. Cancer..

[15] O’Brien RM (2007). A caution regarding rules of thumb for variance inflation factors. Qual Quant..

[16] Reynolds TL, Khan MA, van der Linden S, Cleveland RP (1991). Differences in HLA-B27 positive and negative patients with ankylosing spondylitis: study of clinical disease activity and concentrations of serum IgA, C reactive protein, and haptoglobin. Ann Rheum Dis..

[17] Wendling D (2016). The gut in spondyloarthritis. Jt Bone Spine..

[18] Witte T, et al. Sensitivity and specificity of autoantibodies against CD74 in early axial spondyloarthritis [abstract]. Arthritis Rheumatol. 2016;68(suppl 10). 10.1002/art.40777 30418704

[19] Van Praet L (2014). Degree of bone marrow oedema in sacroiliac joints of patients with axial spondyloarthritis is linked to gut inflammation and male sex: results from the GIANT cohort. Ann Rheum Dis..

[20] Wendling D, Didier JM, Seilles E (1996). Serum secretory immunoglobulins in ankylosing spondylitis. Clin Rheumatol..

[21] Poddubnyy D (2011). Evaluation of 2 Screening Strategies for Early Identification of Patients with Axial Spondyloarthritis in Primary Care. J Rheumatol..

[22] Braun A, Saracbasi E, Grifka J, Schnitker J, Braun J (2011). Identifying patients with axial spondyloarthritis in primary care: how useful are items indicative of inflammatory back pain?. Ann Rheum Dis..

